# Objectively-measured sedentary time and physical activity in a bi-ethnic sample of young children: variation by socio-demographic, temporal and perinatal factors

**DOI:** 10.1186/s12889-019-8132-z

**Published:** 2020-01-28

**Authors:** Paul J. Collings, Sufyan A. Dogra, Silvia Costa, Daniel D. Bingham, Sally E. Barber

**Affiliations:** 1 Bradford Institute for Health Research, Bradford Teaching Hospitals NHS Foundation Trust, Bradford, UK; 2 Department of Health Sciences, University of York, York, UK; 3 School of Sport, Exercise and Health Sciences, Loughborough University, Leicestershire, UK

**Keywords:** Exercise, Sedentary behavior, Pediatrics, Correlates, Determinants, Ethnic groups, Sex, Social class, Obesity, Fetal development

## Abstract

**Background:**

Evidence suggests that South Asian school-aged children and adults are less active compared to the white British population. It is unknown if this generalises to young children. We aimed to describe variability in levels of physical activity and sedentary time in a bi-ethnic sample of young children from a deprived location.

**Methods:**

This observational study included 202 South Asian and 140 white British children aged 1.5 to 5y, who provided 3181 valid days of triaxial accelerometry (Actigraph GT3X+). Variability in sedentary time and physical activity levels were analysed by linear multilevel modelling. Logistic multilevel regression was used to identify factors associated with physical inactivity (failing to perform ≥180 min of total physical activity including ≥60 min moderate-to-vigorous physical activity (MVPA) per day).

**Results:**

There were no significant ethnic differences in the overall levels of behaviours; South Asian and white British children spent half of daily time sedentary, just over 40% in light physical activity, and the remaining 7.5 to 8% of time in MVPA. Sedentary time was lower and physical activity levels were higher in older children, and levels of MVPA and vector magnitude counts per minute (CPM) were higher on weekends compared to weekdays. In South Asian children, sedentary time was lower on weekends. Sedentary time was lower and physical activity levels were higher in spring compared to winter in white British children, and in all seasons compared to winter in South Asian children. South Asian children born at high birth weight performed more MVPA, and in both ethnicities there was some evidence that children with older mothers were more sedentary and less active. Sedentary time was higher and light physical activity was lower in South Asian children in the highest compared to the lowest income families. South Asian girls performed less MVPA, registered fewer vector magnitude CPM, and were 3.5 times more likely to be physically inactive than South Asian boys.

**Conclusions:**

Sedentary time and physical activity levels vary by socio-demographic, temporal and perinatal characteristics in young children from a deprived location. South Asian girls have the most to gain from efforts to increase physical activity levels.

**Trial registration:**

The Pre-schoolers in the Playground (PiP) pilot randomized controlled trial is registered with the ISRCTN (ISRCTN54165860; http://www.isrctn.com).

## Introduction

Physical activity is favourably associated with a multitude of physical, psychosocial and developmental health indicators in young children [1]. Minimising sedentary time in early childhood (in particular TV viewing and other screen-based behaviours) is also important for disease prevention and health promotion [2]. New international guidelines [3–6], which are endorsed by the World Health Organisation [7], continue to recommend that all children younger than 5y who can walk should perform ≥180 min of total physical activity (i.e. at any intensity) every day, but add that children aged 3 to 4y should also accumulate ≥60 min of moderate-to-vigorous physical activity (MVPA). It is consistently recommended that extended periods of sedentary time should be minimised [3–7].

Evidence suggests that differences in sedentary time and physical activity (collectively referred to from hereon as movement behaviours) exist between ethnic groups in the UK, with school-aged children and adults of South Asian origin being less active compared to the white British population [8]. This may partly explain why type 2 diabetes and cardiovascular disease risk is higher in South Asian than white populations in the UK [9]. It is widely accepted that differences in disease risk emerge in early childhood, meaning that early prevention is key [10, 11]. To our knowledge, no research has yet quantified the levels and patterns of movement behaviours performed by young South Asian preschool children relative to their white peers. This is an important knowledge gap to fill as it could highlight when ethnic disparities in movement behaviours start to occur. It could also identify opportunities for prevention or minimisation of ethnic disparities, by facilitating behaviour change interventions that are tailored to the needs of specific ethnic groups [12]. Encouraging healthy and equal habitual movement behaviours from a young age could aid early prevention of ethnic differences in disease risk, and if movement behaviours track across the life-course, could help to reduce longer-term health inequalities [13].

This study aimed to describe the levels and patterns of objectively-measured movement behaviours performed by young South Asian and white British children from a deprived urban setting. We also evaluated adherence to the new international guidelines for physical activity and identified factors associated with failing to meet recommendations [7].

## Methods

Data were pooled from three projects that are related to the Born in Bradford birth cohort study [14] and research programme (www.borninbradford.nhs.uk). Two projects were observational studies [15, 16] and another was a multiple time-point school-based pilot cluster randomised control trial (RCT) that targeted preschool children [17]. Each study included objective measurement of free-living physical activity in young children living in Bradford, which is one of most deprived and ethnically diverse cities in the UK [18]. Data were collected between June 2011 and November 2015. Because intervention attendance was low and there was no evidence for trial effectiveness, all data from both the control and intervention groups of the RCT were used [17]; this allowed each trial participant to contribute up to four repeated measurements of physical activity (collected at baseline, 10, 30 and 52 weeks of follow-up). The study sample used for this investigation was heavily deprived because the RCT recruited schools from the poorest locations in Bradford. Otherwise, there were no differences in the ratio of South Asian to white British participants, or in the distributions of sex, maternal delivery age, and birth weight between this study sample and the population-based Born in Bradford cohort [14]. All studies in this pooled analysis received either National Research Ethics Service or institutional ethical approval. Parental written informed consent and child assent were obtained before measurements.

### Dependent variables: sedentary time and physical activity

Movement data were collected with accelerometers (Actigraph GT3X+, ActiGraph, Florida, USA) worn at the right hip on an elasticated belt for 6–8 consecutive days. Data were sampled at a frequency of 60 Hz, and raw acceleration signals were integrated into 15 s epochs after download into the Actilife software (v6.13, ActiGraph, Florida, USA). Monitor non-wear and daytime napping were inferred from uninterrupted zero activity counts lasting ≥10 min and were discarded. Parent-reported sleep diaries and visual inspection of daily acceleration plots were used to remove overnight sleep. All days with ≥6 h of waking data were considered valid [19] and to mitigate selection biases all children with ≥1 valid day were included in this study. Age-appropriate thresholds were used to estimate the daily proportion of monitor wear time spent sedentary, in light physical activity and MVPA [20]. Total physical activity constituted the sum of light physical activity and MVPA minutes per day. Daily vector magnitude counts per minute (CPM) was used to represent the average physical activity intensity [21]. Each valid day was classified as ‘inactive’ if the new international guideline daily amount of physical activity for 3 to 4y olds had not been met (≥180 min of total physical activity, of which ≥60 min must be MVPA) [7]. Full details of the objective monitoring procedure are available elsewhere [16].

### Independent variables: socio-demographic, temporal and perinatal factors

Child age, sex, ethnicity, and home postcode were gathered from parent reports and school records. Children were classified as South Asian (Pakistani, Bangladeshi or ‘other’ South Asian origin) or white British. Children from other ethnic backgrounds or mixed ancestry were excluded because of small numbers that precluded meaningful analyses (*n* = 41). Home postcodes were grouped according to whether they were among the most deprived 10%, > 10 to 30%, or > 30% of areas relative to the rest of England (lower values represent higher deprivation [22]). Mother’s reported if their personal or household (whichever was most relevant) gross annual income was a) < £6999, b) £7000 to £16,999, c) £17,000 to £25,999, d) £26,000 to £35,000, or e) > £36,000 before tax. Due to small cell numbers (income data were only collected in the RCT) the final three categories were collapsed to form a group representing an annual income before tax of ≥£17,000. Maternal age at delivery was calculated as the time elapsed between the mother and child’s dates of birth and was collapsed to three categories (< 25y, 25 to < 30y, ≥30y). Children who were part of the Born in Bradford birth cohort study were matched to birth weight using NHS patient identifiers [23]. Birth weight was categorised as low (< 2500 g), normal (2500 to 3500 g), or high (> 3500 g) [24]. Time-stamped information from accelerometers were used to classify weekdays (Monday to Friday) and weekends (Saturday and Sunday) and also to assign the season of measurement (winter: December to February; spring: March to May; summer: June to August; autumn: September to November). Child height and weight were measured by trained researchers using standard procedures and calibrated equipment [16]. The data were used to calculate body mass index (BMI, kg/m^2^) which was converted to *z*-scores and weight status categories [25].

### Statistical analysis

Descriptive characteristics were summarised for the total sample and stratified by ethnicity. Participant level characteristics were compared between South Asian and white British children using chi-square tests. Age and weight status were measured at each time-point and were compared between ethnic groups using linear and ordered logistic regression, respectively, with a random intercept to account for repeated-measurements clustered within children. Daily level data were compared between ethnic groups using linear and logistic regressions, but with the addition of another random intercept to account for days nested within measurement time-points, further clustered within children.

The main analysis was preceded by creating multiple (*n* = 60) imputed datasets by chained equations [26] to account for missing data for maternal delivery age (missing for 27.2% of children), annual income (61.1%), birth weight (62.6%), and weight status (missing for 18.7% of time-points). Imputation was carried out under a missing at random assumption and included all variables involved in the analysis. Linear multilevel models were thereafter used to quantify the levels and patterns of sedentary time and physical activity (separate models for each dependent variable). Models were specified with random intercepts to account for valid days of accelerometry (level 1) nested within measurement time-points (level 2) clustered within children (level 3). There was limited variation in outcomes at the school and study level so accounting for higher-order clustering was unnecessary; this was confirmed by likelihood ratio tests for model fit. Models were specified with ethnicity, sex, age (modelled continuously), area deprivation, weight status, type of day, season, birth weight, and maternal delivery age simultaneously included as independent variables to mutually adjustment for each other. Models were subsequently rerun with annual income as an independent variable instead of area deprivation. Interaction terms between ethnicity and all potential correlates (ethnicity*correlate) were added sequentially and likelihood ratio tests were used to examine improved model fit. The results are presented as estimated marginal means with 95% confidence intervals (CI). Due to positive skewness, MVPA was natural log transformed prior to analyses, the results have been back-transformed for reporting. To identify factors related to the likelihood of inactivity (failing to perform ≥180 min of total physical activity, including ≥60 min MVPA) multilevel logistic regression models were used to calculate odds ratios (OR). Logistic models were specified identically to linear models. To ensure robustness in the results a complete-case analysis was performed. In addition, all analyses were replicated after excluding all non-baseline data from children who were in the intervention group of the RCT. A significance level of *p* < 0.05 was chosen a priori and all analyses were performed with Stata/SE 13.1 software (StataCorp, College Station, TX).

## Results

**Fig. 1:**
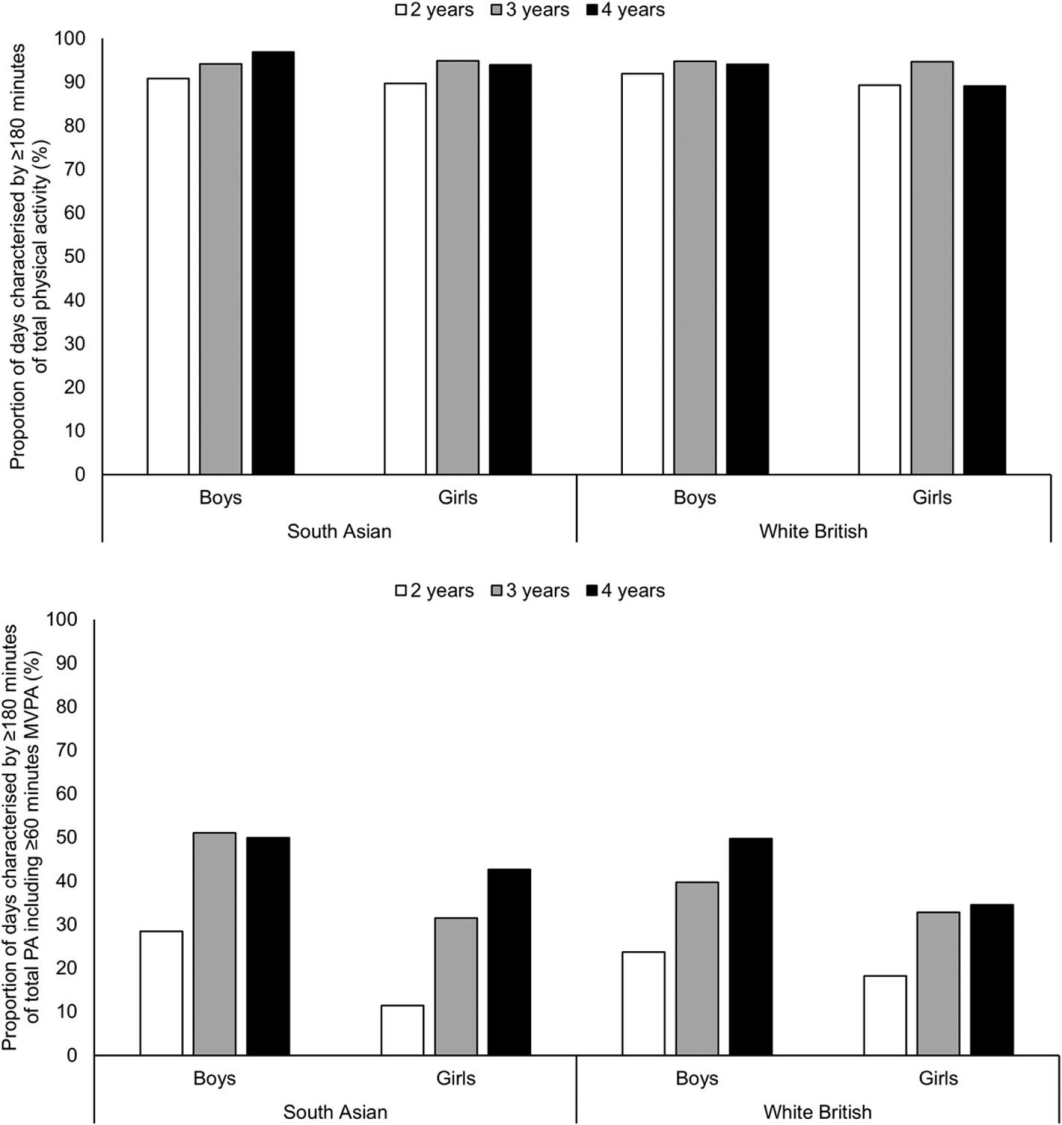
Proportion of days characterised by meeting the former guideline daily amount of physical activity for all children aged younger than 5 years (≥180 min of total physical activity) and the new international guideline daily amount of physical activity for children aged 3 to 4 years (≥180 min of total physical activity including ≥60 min MVPA), stratified by ethnicity, gender and age. The top tile relates to meeting the guideline daily amount of physical activity for all children aged younger than 5 years who are capable of walking. The bottom tile relates to meeting the new international guideline daily amount of physical activity for children aged 3 to 4 years. Sample sizes: South Asian boys aged 2 years: 27 children; 64 time-points; 260 days; South Asian boys aged 3 years: 43 children; 72 time-points; 339 days; South Asian boys aged 4 years: 30 children; 42 time-points; 323 days; South Asian girls aged 2 years: 36 children; 93 time-points; 300 days; South Asian girls aged 3 years: 37 children; 74 time-points; 461 days; South Asian girls aged 4 years: 29 children; 35 time-points; 328 days; white British boys aged 2 years: 26 children; 45 time-points; 186 days; white British boys aged 3 years: 41 children; 68 time-points; 320 days; white British boys aged 4 years: 8 children; 8 time-points; 133 days; white British girls aged 2 years: 27 children; 48 time-points; 214 days; white British girls aged 3 years: 32 children; 45 time-points; 262 days; white British girls aged 4 years: 6 children; 6 time-points; 55 days. Due to small numbers, for 7 children aged 1.6 to 1.9 years (contributing 12 time-points and 139 days), age was rounded to 2 years. For another 7 children aged 5.0 to 5.1 years (contributing 7 time-points and 67 days) age was rounded to 4 years. PA, physical activity. MVPA, moderate-to-vigorous physical activity

The final sample included 342 children (59.1% South Asian; 51.2% boys) measured at 600 time-points, and who contributed in total 3181 valid days (totalling 32,000 h) of data. Table 1 provides a description of the sample. Nearly two-thirds of children lived in the most deprived 10% of locations in England, with a higher proportion of South Asian than white British children living in the most deprived areas. A higher proportion of South Asian than white British children were from families with an annual income of £7000 to 16,999. Fewer South Asian children were born to the youngest mothers and had a high birth weight. Nearly half of children were measured more than once, with children participating at two (18.4%), three (19.3%), and four (6.1%) time-points. In total, children contributed on average 7 valid days of accelerometry (range: 1 to 31 days). One-third of children were measured in two different seasons and nearly 10% contributed three seasons of measurement. On average, children were 3.4y (range: 1.6 to 5.1y) of age at the time of measurements, and nearly one-fifth of children were overweight and obese. Overweight and obesity were more prevalent in white British children. Average daily wear time exceeded 10 h, one-quarter of days were weekends, and there was reasonably even distribution of days across seasons. Overall, the vast majority of days (93.3%) were characterised by ≥180 min of total physical activity, which until recently has been the recommended daily amount for all children aged younger than 5y. In contrast, only 34.8% of days were characterised by ≥180 min of total physical activity inclusive of ≥60 min MVPA, which is the new international guideline for children aged 3 to 4y. Fig. 1 shows the proportion of days that were characterised by meeting the former and the new international guidelines, stratified by ethnicity, gender and age.

**Table 1:** Descriptive characteristics of the study population

Data level	Characteristic	Total (*n* = 342)	South Asian (*n* = 202)	White British (*n* = 140)	*p*-value ethnic difference
Participant	Sex (*n* (%))				
	Boys	175 (51.2)	100 (49.5)	75 (53.6)	
	Girls	167 (48.8)	102 (50.5)	65 (46.4)	0.46
	Area deprivation^a^ (*n* (%))				
	Most deprived 10%	209 (61.1)	146 (72.3)	63 (45.0)	
	> 10 to 30%	94 (27.5)	46 (22.8)	48 (34.3)	
	> 30%	39 (11.4)	10 (4.9)	29 (20.7)	< 0.001
	Annual income (*n* (%))				
	<£6999	36 (27.1)	19 (22.9)	17 (34.0)	
	£7000 to £16,999	60 (45.1)	46 (55.4)	14 (28.0)	
	≥£17,000	37 (27.8)	18 (21.7)	19 (38.0)	0.008
	Maternal delivery age (*n* (%))				
	< 25y	74 (29.7)	26 (20.2)	48 (40.0)	
	≥25 to < 30	98 (39.4)	60 (46.5)	38 (31.7)	
	≥30y	77 (30.9)	43 (33.3)	34 (28.3)	0.002
	Birth weight (*n* (%))				
	Low	10 (7.8)	7 (10.8)	3 (4.8)	
	Normal	77 (60.2)	44 (67.7)	33 (52.4)	
	High	41 (32.0)	14 (21.5)	27 (42.9)	0.026
		Total (*n* = 600)	South Asian (*n* = 380)	White British (*n* = 220)	
Time-point	Age (y)	3.4 ± 0.8	3.4 ± 0.9	3.2 ± 0.7	< 0.001
	Weight status^b^ (*n* (%))				
	Underweight	23 (4.7)	19 (5.9)	4 (2.4)	
	Healthy weight	371 (76.0)	251 (77.4)	120 (73.2)	
	Overweight or obese	94 (19.3)	54 (16.7)	40 (24.4)	0.014
		Total (*n* = 3181)	South Asian (*n* = 2011)	White British (*n* = 1170)	
Daily	Wear time (min/d)	603.8 (199.3)	604.3 (208.3)	601.9 (181.8)	0.051
	Type of day (*n* (%))				
	Weekday	2418 (76.0)	1524 (75.8)	894 (76.4)	
	Weekend	763 (24.0)	487 (24.2)	276 (23.6)	0.69
	Season (*n* (%))				
	Winter	647 (20.3)	365 (18.2)	282 (24.1)	
	Spring	729 (22.9)	404 (20.1)	325 (27.8)	
	Summer	609 (19.1)	380 (18.9)	229 (19.6)	
	Autumn	1196 (37.6)	862 (42.9)	334 (28.6)	< 0.001
	Inactive (*n* (%))^c^	2073 (65.2)	1284 (63.9)	789 (67.4)	0.21

Ethnic differences were analysed using chi-square tests at the participant level; linear and ordered logistic regression with a random intercept to account for clustering within children at the time-point level; linear and ordered logistic regression with two random intercepts to account for days nested within measurement time-points clustered within children at the daily level. Wear time was skewed and was natural log transformed prior to analysis, and median (iqr) rather than mean ± standard deviation values are presented. ^a^Based on the national measure of relative deprivation for small areas in England. ^b^Based on British growth reference data. ^c^Inactive was defined as failing to meet the new international guideline daily amount of physical activity for children aged 3 to 4 years (≥180 min of total physical activity including ≥60 min MVPA). South Asian ethnicity includes Pakistani (*n* = 154), Bangladeshi (*n* = 21), Indian (*n* = 4), and ‘Other South Asian’ including specific country of origin unknown (*n* = 23). MVPA, moderate-to-vigorous physical activity

Table 2 summarises the daily levels and patterns of sedentary time and physical activity. There were no ethnic differences in the overall levels of behaviours, with both South Asian and white British children spending half of their time sedentary, just over 40% in light physical activity, and the remaining 7.5 to 8% of daily time in MVPA. Post-hoc gender-stratified analyses did reveal, however, some evidence that South Asian boys were less sedentary (*p* = 0.092), performed more MVPA (*p* = 0.052) and registered more vector magnitude CPM compared to white British boys (*p* = 0.062); there was no evidence for ethnic differences in girls (*p* ≥ 0.78). Interaction terms between ethnicity and all potential correlates (ethnicity*correlate) highlighted that patterns of movement behaviours consistently differed between South Asian and white British children. All subsequent analyses were therefore stratified by ethnic group. South Asian girls performed less MVPA and registered fewer vector magnitude CPM than South Asian boys; there were no such gender differences in white British children. Sedentary time did not vary by gender in either ethnic group. In both ethnicities, sedentary time was lower and physical activity levels were higher with increasing age, and levels of MVPA and vector magnitude CPM were higher on weekends than weekdays. Sedentary time was lower on weekends than weekdays only in South Asian children. In white British children, sedentary time was lower and physical activity levels were higher in spring than winter. White British children also performed more light physical activity in summer and there was some evidence that white British children were less sedentary in summer than winter. In South Asian children, sedentary time was lower and physical activity levels were consistently higher in all seasons compared to winter. South Asian children of high birth weight performed more MVPA than their normal birth weight counterparts. There was also some evidence that South Asian children with the oldest mothers were more sedentary, performed less MVPA and registered fewer vector magnitude CPM, compared to South Asian children with the youngest mothers. White British children with mothers in the middlemost age group (aged ≥25 to < 30y at delivery) were more sedentary and performed less light physical activity. There was also some indication that white British children with the oldest mothers accumulated fewer vector magnitude CPM. There was no evidence in either ethnic group that movement behaviours varied by area deprivation, but South Asian children in the highest compared to the lowest income households were more sedentary and performed less light physical activity.

**Table 2:** Daily levels and patterns of physical activity and sedentary time

	Characteristic	Sedentary time (%)	*p*-value	Light activity (%)	*p*-value	MVPA (%)	*p*-value	Vector Magnitude (CPM)	*p*-value
South Asian (participants: 202; time-points: 380; 2011 days)	Overall	48.6 (47.6 to 49.7)	Ref	42.2 (41.4 to 43.0)	Ref	8.0 (7.6 to 8.6)	Ref	1392.9 (1353.3 to 1432.4)	Ref
	Sex								
	Boys	48.2 (46.8 to 49.5)	Ref	41.8 (40.7 to 42.8)	Ref	8.9 (8.2 to 9.7)	Ref	1444.2 (1391.7 to 1496.6)	Ref
	Girls	49.5 (48.2 to 50.8)	0.18	42.3 (41.3 to 43.3)	0.46	**7.1 (6.6 to 7.7)**	**< 0.001**	**1330.3 (1279.8 to 1380.7)**	**0.003**
	Age (y)								
	2	**50.7 (48.8 to 52.5)**		41.7 (40.3 to 43.2)		**6.5 (5.8 to 7.3)**		**1285.7 (1213.8 to 1357.5)**	
	3	**49.5 (48.4 to 50.5)**	*p*-trend	42.0 (41.1 to 42.8)	*p*-trend	**7.4 (7.0 to 7.9)**	*p*-trend	**1351.2 (1311.3 to 1391.1)**	*p*-trend
	4	**48.3 (47.1 to 49.4)**	**0.036**	42.2 (41.3 to 43.0)	0.63	**8.4 (7.9 to 9.0)**	**< 0.001**	**1416.8 (1375.0 to 1458.5)**	**0.003**
	Area deprivation								
	Most deprived 10%	49.1 (48.0 to 50.2)	Ref	41.9 (41.0 to 42.7)	Ref	7.9 (7.4 to 8.4)	Ref	1377.8 (1337.5 to 1418.1)	Ref
	> 10 to 30%	47.6 (45.5 to 49.8)	0.23	42.9 (41.3 to 44.6)	0.24	8.2 (7.2 to 9.2)	0.61	1418.8 (1338.3 to 1499.3)	0.38
	> 30%	50.1 (46.0 to 54.2)	0.65	41.6 (38.5 to 44.8)	0.89	7.4 (5.9 to 9.3)	0.60	1324.8 (1172.8 to 1476.9)	0.51
	Annual income (£)								
	<£6999	47.9 (45.8 to 50.0)	Ref	43.2 (41.4 to 45.0)	Ref	7.9 (7.2 to 8.8)	Ref	1389.2 (1320.6 to 1457.9)	Ref
	£7000 to £16,999	48.5 (46.9 to 50.1)	0.71	42.1 (40.9 to 43.3)	0.37	8.2 (7.5 to 9.0)	0.62	1405.0 (1345.3 to 1464.6)	0.76
	≥£17,000	**51.5 (48.9 to 54.0)**	**0.050**	**40.0 (38.0 to 42.0)**	**0.032**	7.1 (6.1 to 8.2)	0.24	1323.2 (1229.2 to 1417.2)	0.29
	Weight status								
	Underweight	49.5 (45.7 to 53.3)	0.72	41.1 (38.2 to 44.0)	0.52	8.0 (6.3 to 10.1)	0.89	1398.7 (1261.0 to 1536.4)	0.84
	Healthy weight	48.8 (47.8 to 49.9)	Ref	42.1 (41.3 to 42.9)	Ref	7.9 (7.4 to 8.4)	Ref	1384.3 (1345.3 to 1423.3)	Ref
	Overweight / obese	49.0 (46.7 to 51.3)	0.88	42.2 (40.4 to 44.0)	0.95	7.9 (6.9 to 9.0)	0.99	1367.9 (1284.2 to 1451.7)	0.73
	Type of day								
	Weekday	49.2 (48.3 to 50.2)	Ref	42.0 (41.3 to 42.7)	Ref	7.7 (7.3 to 8.1)	Ref	1360.2 (1325.1 to 1395.3)	Ref
	Weekend	**47.7 (46.7 to 48.8)**	**< 0.001**	42.3 (41.5 to 43.1)	0.27	**8.7 (8.2 to 9.3)**	**< 0.001**	**1452.2 (1412.7 to 1491.8)**	**< 0.001**
	Season								
	Winter	52.4 (50.6 to 54.2)	Ref	39.4 (38.0 to 40.9)	Ref	6.9 (6.2 to 7.8)	Ref	1262.5 (1197.2 to 1327.8)	Ref
	Spring	**48.4 (46.5 to 50.4)**	**0.002**	**42.1 (40.6 to 43.6)**	**0.008**	**8.3 (7.4 to 9.3)**	**0.025**	**1413.3 (1342.9 to 1483.6)**	**0.001**
	Summer	**47.2 (45.5 to 48.8)**	**< 0.001**	**43.3 (42.0 to 44.6)**	**< 0.001**	**8.5 (7.7 to 9.4)**	**0.004**	**1444.9 (1385.2 to 1504.6)**	**< 0.001**
	Autumn	**48.3 (47.0 to 49.6)**	**< 0.001**	**42.6 (41.6 to 43.6)**	**< 0.001**	7.9 (7.3 to 8.5)	0.051	**1391.3 (1342.1 to 1440.5)**	**< 0.001**
	Birth weight								
	Low	49.7 (46.5 to 52.9)	0.83	42.1 (39.6 to 44.6)	0.86	7.3 (6.0 to 8.8)	0.65	1315.1 (1195.7 to 1434.5)	0.46
	Normal	49.3 (47.8 to 50.7)	Ref	41.9 (40.7 to 43.0)	Ref	7.6 (7.0 to 8.3)	Ref	1366.4 (1312.8 to 1419.9)	Ref
	High	46.7 (43.3 to 50.0)	0.17	42.7 (40.4 to 45.1)	0.51	**9.6 (7.8 to 11.7)**	**0.046**	1510.1 (1371.1 to 1649.1)	0.062
	Maternal delivery age (y)								
	< 25	47.7 (45.4 to 50.1)	Ref	42.9 (41.1 to 44.8)	Ref	8.2 (7.2 to 9.4)	Ref	1414.6 (1328.1 to 1501.1)	Ref
	≥25 to < 30	48.0 (46.5 to 49.5)	0.87	42.4 (41.2 to 43.5)	0.61	8.6 (7.9 to 9.4)	0.60	1429.4 (1371.5 to 1487.3)	0.78
	≥30	50.5 (48.8 to 52.2)	0.069	41.3 (40.0 to 42.6)	0.16	7.0 (6.4 to 7.7)	0.057	1313.3 (1251.4 to 1375.2)	0.071
White British (participants: 140; time-points: 220; days: 1170)	Overall	49.8 (48.5 to 51.2)	0.22	41.5 (40.4 to 42.5)	0.28	7.5 (6.9 to 8.1)	0.22	1349.5 (1297.7 to 1401.4)	0.24
	Sex								
	Boys	49.7 (48.0 to 51.4)	Ref	41.0 (39.8 to 42.3)	Ref	8.0 (7.2 to 8.9)	Ref	1381.1 (1321.5 to 1440.7)	Ref
	Girls	48.9 (47.0 to 50.7)	0.55	42.6 (41.2 to 44.0)	0.10	7.4 (6.6 to 8.3)	0.28	1355.7 (1290.4 to 1421.1)	0.59
	Age (y)								
	2	**52.8 (50.6 to 55.0)**		**40.1 (38.5 to 41.8)**		**6.0 (5.3 to 6.9)**		**1217.7 (1141.3 to 1294.1)**	
	3	**49.9 (48.7 to 51.1)**		**41.5 (40.6 to 42.4)**		**7.4 (6.9 to 8.0)**		**1344.4 (1301.1 to 1387.7)**	
	4	**47.0 (45.2 to 48.7)**	**< 0.001**	**42.9 (41.5 to 44.2)**	**0.027**	**9.1 (8.1 to 10.2)**	**< 0.001**	**1471.1 (1408.5 to 1533.8)**	**< 0.001**
	Area deprivation								
	Most deprived 10%	49.4 (47.5 to 51.2)	Ref	41.8 (40.4 to 43.2)	Ref	7.7 (6.9 to 8.7)	Ref	1365.5 (1300.2 to 1430.7)	Ref
	> 10 to 30%	49.6 (47.4 to 51.7)	0.91	41.7 (40.1 to 43.4)	0.97	7.4 (6.5 to 8.5)	0.69	1360.6 (1284.1 to 1437.0)	0.93
	> 30%	48.9 (46.1 to 51.6)	0.76	41.8 (39.7 to 43.9)	0.99	8.2 (6.9 to 9.8)	0.56	1391.3 (1294.5 to 1488.1)	0.67
	Annual income (£)								
	<£6999	48.9 (46.6 to 51.1)	Ref	42.2 (40.5 to 44.0)	Ref	7.4 (6.4 to 8.7)	Ref	1383.9 (1302.4 to 1465.5)	Ref
	£7000 to £16,999	50.0 (46.5 to 53.5)	0.60	41.5 (38.9 to 44.1)	0.65	7.3 (5.8 to 9.2)	0.92	1337.3 (1213.3 to 1461.4)	0.55
	≥£17,000	49.5 (47.5 to 51.5)	0.70	41.4 (39.9 to 43.0)	0.54	8.1 (7.1 to 9.3)	0.47	1367.8 (1296.2 to 1439.4)	0.79
	Weight status								
	Underweight	51.3 (43.7 to 58.8)	0.68	41.3 (35.6 to 46.9)	0.94	5.7 (3.0 to 10.9)	0.37	1254.6 (992.1 to 1517.1)	0.45
	Healthy weight	49.7 (48.2 to 51.1)	Ref	41.5 (40.4 to 42.6)	Ref	7.7 (7.0 to 8.4)	Ref	1357.2 (1306.5 to 1407.9)	Ref
	Overweight / obese	48.2 (45.8 to 50.7)	0.32	42.5 (40.7 to 44.4)	0.34	8.0 (6.8 to 9.5)	0.66	1413.1 (1325.4 to 1500.7)	0.29
	Type of day								
	Weekday	49.4 (48.2 to 50.6)	Ref	41.9 (41.0 to 42.8)	Ref	7.6 (7.0 to 8.2)	Ref	1356.2 (1313.0 to 1399.4)	Ref
	Weekend	49.2 (47.7 to 50.6)	0.68	41.3 (40.2 to 42.4)	0.17	**8.3 (7.6 to 9.1)**	**< 0.001**	**1412.8 (1361.9 to 1463.6)**	**0.002**
	Season								
	Winter	51.0 (49.0 to 53.0)	Ref	40.6 (39.0 to 42.2)	Ref	7.1 (6.2 to 8.1)	Ref	1313.3 (1242.8 to 1383.9)	Ref
	Spring	**48.3 (46.3 to 50.3)**	**0.039**	42.2 (40.7 to 43.7)	0.10	**8.5 (7.5 to 9.6)**	**0.034**	**1408.4 (1340.0 to 1476.9)**	**0.034**
	Summer	48.6 (46.5 to 50.8)	0.098	**42.9 (41.3 to 44.6)**	**0.033**	7.5 (6.6 to 8.6)	0.54	1382.1 (1307.8 to 1456.4)	0.16
	Autumn	49.4 (47.4 to 51.3)	0.19	41.5 (40.0 to 43.0)	0.35	7.8 (6.9 to 8.8)	0.26	1370.6 (1303.0 to 1438.3)	0.18
	Birth weight								
	Low	49.5 (42.8 to 56.3)	0.89	42.0 (37.0 to 47.0)	0.81	6.8 (4.3 to 10.9)	0.74	1356.2 (1114.7 to 1597.7)	0.90
	Normal	50.0 (47.9 to 52.1)	Ref	41.3 (39.8 to 42.9)	Ref	7.4 (6.5 to 8.5)	Ref	1340.0 (1263.2 to 1416.9)	Ref
	High	48.5 (46.1 to 51.0)	0.42	42.2 (40.4 to 43.9)	0.53	8.2 (7.1 to 9.6)	0.35	1403.6 (1311.6 to 1495.7)	0.37
	Maternal delivery age (y)								
	< 25	48.0 (46.0 to 50.0)	Ref	42.7 (41.2 to 44.1)	Ref	8.3 (7.3 to 9.3)	Ref	1425.6 (1357.3 to 1493.8)	Ref
	≥25 to < 30	50.8 (48.5 to 53.1)	0.068	**40.3 (38.5 to 42.1)**	**0.047**	7.4 (6.4 to 8.6)	0.26	1338.7 (1256.3 to 1421.1)	0.12
	≥30	49.8 (47.5 to 52.1)	0.24	41.8 (40.0 to 43.5)	0.46	7.4 (6.4 to 8.6)	0.30	1327.6 (1244.0 to 1411.2)	0.086

Statistical analyses performed on multiple (*n* = 60) imputed datasets using multilevel linear regression to account for clustering of days within time-points and children. Overall estimates are from analyses of the total sample with ethnic group modelled as an independent variable, *p*-values represent differences compared to the South Asian ethnic group. Otherwise all analyses were stratified by South Asian (202 children; 380 time-points; 2011 days) and white British (140 children; 220 time-points; 1170 days) ethnic groups. All factors were mutually adjusted for one another except area deprivation and annual income which occupied separate models. South Asian ethnicity includes Pakistani (*n* = 154), Bangladeshi (*n* = 21), Indian (*n* = 4), and ‘Other South Asian’ including country of origin unknown (*n* = 23). MVPA, moderate-to-vigorous physical activity

**Fig. 2:**
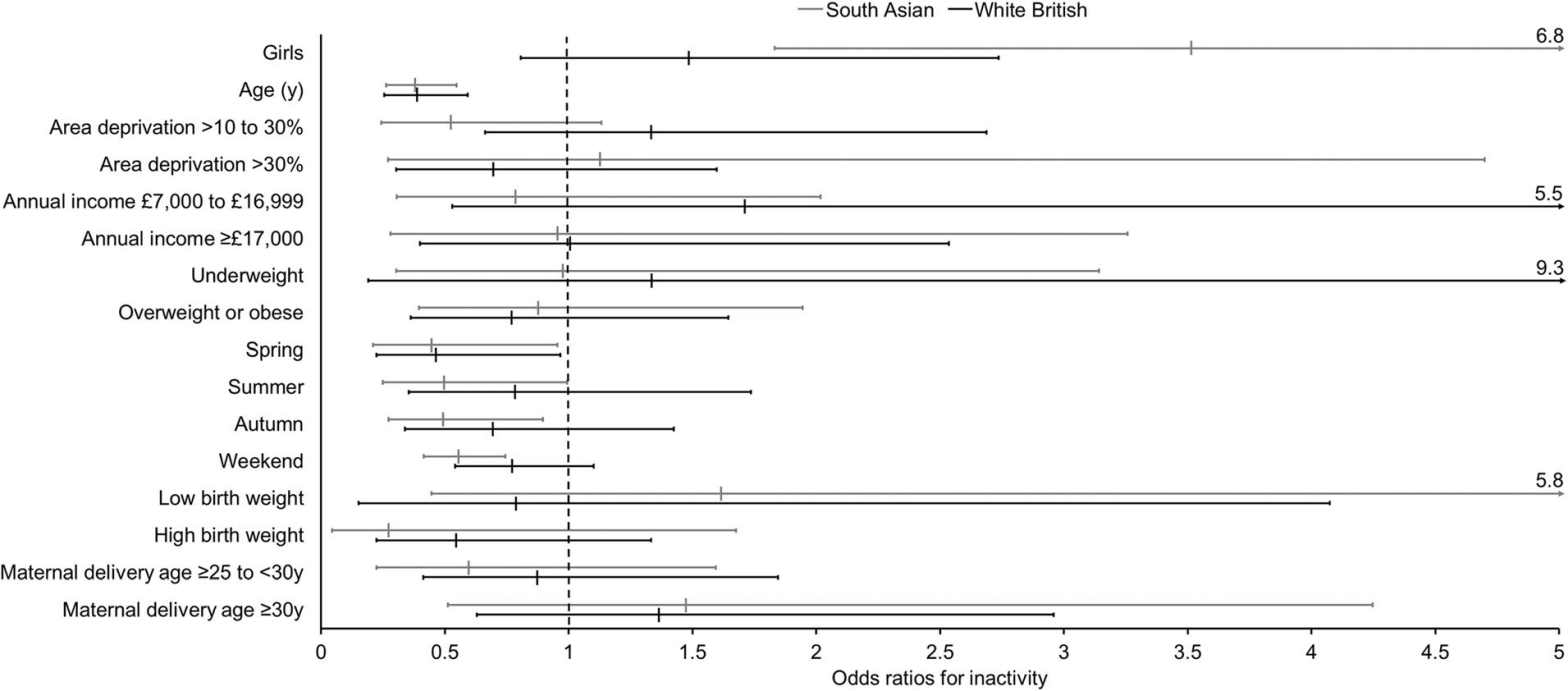
Odds ratios for failing to meet the new international guideline daily amount of physical activity for children aged 3 to 4 years (≥180 min of total physical activity including ≥60 min MVPA). Statistical analyses performed on multiple (*n* = 60) imputed datasets using multilevel logistic regression to account for clustering of days within time-points and children. Analyses were stratified by South Asian (202 children; 380 time-points; 2011 days) and white British (140 children; 220 time-points; 1170 days) ethnicities. All factors were mutually adjusted for one another except area deprivation and annual income which occupied separate models from each other. Reference categories are boys, area deprivation ≤10%, annual income <£6999, healthy weight, winter, weekday, normal birth weight, and maternal delivery age < 25y, respectively. Age was modelled continuously. South Asian ethnicity includes Pakistani (*n* = 154), Bangladeshi (*n* = 21), Indian (*n* = 4), and ‘Other South Asian’ including country of origin unknown (*n* = 23). MVPA, moderate-to-vigorous physical activity

On two-thirds of days children were classified as inactive as they did not meet the new international guideline daily amount of physical activity for 3 to 4y olds (≥180 min of total physical activity, including ≥60 min MVPA). Results quantifying the likelihood of physical inactivity are presented in Fig. 2. Overall, there was no difference between South Asian and white British children in the likelihood of being physically inactive (OR = 1.24 95% CI 0.72 to 2.14, *p* = 0.45 for white British versus South Asian as the reference group). Post-hoc gender-stratified analyses did reveal, however, some evidence that white British boys were more likely to be inactive compared to South Asian boys (OR = 1.85 95% CI 0.90 to 3.82, *p* = 0.092); there was no comparable evidence in girls (OR = 0.70 95% CI 0.30 to 1.64, *p* = 0.41). In both ethnic groups, each year of age was associated with ~ 60% lower odds of physical inactivity (South Asian: OR = 0.38, 95% CI 0.26 to 0.55, *p* < 0.001; white British: OR = 0.39, 95% CI 0.25 to 0.59, *p* < 0.001). White British children were 54% less likely to be physically inactive in spring than winter (OR = 0.46, 95% CI 0.22 to 0.97, *p* = 0.040), and South Asian children were approximately 50% less likely to be physically inactive in all seasons compared to winter (spring: OR = 0.45, 95% CI 0.21 to 0.96, *p* = 0.038; summer: OR = 0.50, 95% CI 0.25 to 0.99, *p* < 0.048; autumn: OR = 0.49, 95% CI 0.27 to 0.90, *p* < 0.020). South Asian children were 44% less likely to be physically inactive on weekends than weekdays (OR = 0.56, 95% CI 0.41 to 0.74, *p* < 0.001). Finally, South Asian girls were 3.5 times more likely to be physically inactive compared to South Asian boys (OR = 3.5, 95% CI 1.8 to 6.7, *p* < 0.001). There were no substantive differences in any of the results in complete-case analyses, or analyses that excluded all non-baseline data from children who were in the intervention group of the RCT.

## Discussion

### Levels of sedentary time and physical activity

This is the first study to show that young South Asian and white British children from a deprived urban location spend half of their daily time sedentary, just over 40% in light physical activity, and remaining time in MVPA. Wide variability exists in the levels of objectively measured movement behaviours in young children [27]. In part these differences are due to methodological inconsistencies, including different accelerometer types and placement, data reduction and processing, all of which prevent a direct (like-for-like) comparison of results across study populations. Even so, our results appear to be similar to studies that have incorporated the same accelerometer, device placement and cutpoints in children of comparable ages from the Netherlands [28], Sweden [29], Switzerland [30] and America [31]; although the children in the current study participated in slightly more sedentary time and less light physical activity. Another study conducted in England, by Hesketh and colleagues, used a different measurement procedure and was performed in a predominantly white population from a smaller and more affluent city in the South of the country. That study found that children performed a similar amount of MVPA, but nearly 60% of daily time was spent in light physical activity and only one-third was sedentary time [32]. The estimate provided by Hesketh et al. differs considerably from a recent meta-analysis which calculated that children aged 2-6y spend 51.4% (95% CI: 48.4 to 54.4%) of daily time sedentary [33]. This concurs with our novel results in young South Asian and white British children, who also spent approximately half of daily time in sedentary behaviour.

### Patterns of sedentary time and physical activity

This is the first study to compare the levels and patterns of movement behaviours in young South Asian and white British children. Similar studies have been performed in school-aged children (most of them using data more than a decade old) and they have consistently found that South Asian children are less physically active compared to white British children [8, 34]. This was not observed in the current sample of young children. Ultimately, we found no significant ethnic differences in levels of sedentary time or physical activity, even though there was some indication that South Asian boys were less sedentary and more active than white British boys. This could indicate that meaningful ethnic differences only manifest when children are older, which would imply that early prevention is key to preventing ethnic inequalities in physical activity. Alternatively, second generation UK South Asians are more likely to be educated beyond school [35], and they purportedly have different attitudes to physical activity and are more active compared to the first (migrant) generation [8]. Plausibly, as knowledge, attitudes and behaviours in the youngest generation of UK South Asians and their UK born parents have continued to change, this may have translated to parity in children’s physical activity levels compared to the white British. Adding support to this hypothesis, we recently reported that there were no differences in the levels of light physical activity or MVPA performed by present-day South Asian and white British primary school children aged 6 to 8y [36]. New studies of contemporary datasets are warranted to revisit the presumption that South Asian children are less active than white British children.

Sedentary time did not significantly vary by ethnicity in this study of young children, but it is unfortunate that we were not able to scrutinise different types of sedentary behaviour. We have previously shown that compared to white British children, Pakistani infants watch more television [37], which is associated with myriad adverse health outcomes including obesity [38]. South Asian primary school children have consistently been shown as more sedentary compared to white British children [36, 39]. This is speculated to be because of Madrassa, an after-school religious commitment that starts around age 5y for South Asian children [40, 41]. Most children in this study were measured when they were younger than 5y and hence we may have missed this transition, because we found that sedentary time decreased with advancing age in both ethnic groups, and levels of MVPA and vector magnitude CPM increased. This spike in MVPA (which precedes an age-related decline throughout the life-course [42]) has been reported previously and is often attributed to young children mastering movement skills [43, 44]. We observed that older white British children performed more light physical activity than younger white British children, but there were no such differences in South Asian children. This could be meaningful for future health inequalities if the trend was to continue, because light physical activity is associated with lower adiposity and higher aerobic fitness in mid-childhood [45].

A recent review and meta-analysis reported that young boys are more physically active [46] and less sedentary [33] than young girls, but there are several large and methodologically sound studies that report no differences by sex (e.g. [47–49]). We found no differences in movement behaviours between white British boys and girls. This is in contrast to Hesketh et al., who found that white British boys accumulated more MVPA than girls [32]. The difference in results could be explained by incomparable measurement procedures, combined with the already described differences between study populations. Although we found no gender differences in white British children, South Asian boys performed more MVPA and registered more vector magnitude CPM than South Asian girls. Islamic religious settings like mosques or madrassa, with support and training from local authorities to organise and encourage physical activity, could provide important opportunities to reduce the observed gender differences in South Asian children [50].

It is uncertain whether physical activity levels differ between week and weekend days in young children [46]. We found that MVPA and vector magnitude CPM were higher on weekends in both ethnic groups. In white British children of comparable age, Hesketh et al. found that overall levels of light physical activity and MVPA did not vary between weekdays and weekends, but there were differences between particular segments of days (e.g. children performed more MVPA on weekend afternoons and evenings compared to weekdays) [32]. With regard to sedentary time, our results for white British children concur with meta-analysed results from four studies of young children, which together indicated there were no differences in sedentary time by type of day [33]. We did uniquely observe, however, that sedentary time was lower on weekends than weekdays in South Asian children.

There is inconsistent evidence for seasonal differences in the physical activity of young children [46]. We found that white British children were less sedentary and more physically active in spring compared to winter, and to some extent they were less sedentary and more light physically active in summer than winter. South Asian children were markedly less sedentary and more physically active in all seasons relative to winter. This suggests that the movement behaviours of young South Asian children are markedly hampered in winter, more so than their white British counterparts. This could be due to a combination of cultural and environmental differences. It has been suggested that South Asian parents prefer to keep children indoors given inclement weather or winter colds [17, 41]. Additionally, the South Asian families who took part in this study lived in the most deprived areas. They may have had less access to quality indoor play areas and more concerns about neighbourhood and traffic safety on dark winter evenings [51].

A recent review found that there was no evidence in young children for associations of parental education or other markers of socio-economic status with physical activity [46]. Aside from parental education, geography based measures of neighbourhood deprivation have most commonly been used to signify socio-economic status. In line with earlier studies, we found no evidence that movement behaviours varied by area deprivation. It is possible, however, that an area level marker of socio-economic status may have introduced misclassification errors and biased associations to the null [52]. Supporting this assertion, we found that household income varied markedly within each group of area deprivation (e.g., in the most deprived 10% of areas the distribution of household income was <£6999 (30.7%), £7000 to 16,999 (50.5%) and ≥ £17,000 (18.8%)). With this in mind, it is valuable that we were also able to investigate household income as an individual level marker of socio-economic status. Consistent with the results for area deprivation, no associations were apparent in white British children, but South Asian children in the highest compared to the lowest income households were more sedentary and performed less light physical activity. We have previously reported that TV viewing is prevalent in young South Asian children who watch more TV overall [37] and in the evening than white British children [38]. It is plausible that South Asian children in higher than lower income households may have more access to TVs and other screen-based electronic media (including laptops, tablets, games consoles, and smartphones, equipment which can be prohibitively expensive), thereby encouraging more sedentary time and displacing light physical activity. Unlike physical activity participation in older children, which can require sports equipment or fees for club membership, physical activity performed by young children is mostly informal and play-based and rarely incurs financial cost [53]. This might explain why levels of MVPA in our sample of young children did not vary by household income.

In young children there is inconsistent evidence for associations between physical activity with adiposity [1] and associations for sedentary behaviours are predominantly null [2]. It is a limitation, however, that the majority of studies have used BMI as a proxy measure of adiposity. This may also explain why we failed to find evidence of variation in movement behaviours by weight status. We have previously reported (in a study that consisted largely of the same sample of children) dose-dependent associations between physical activity intensity with lower sum of skinfolds, a more direct marker of adiposity [16]. It is unfortunate that the diminutive number of underweight children in the current analysis precludes any meaningful inference for this particular category. Very low levels of physical activity have previously been observed in young Ethiopian children with severe acute malnutrition [54]. Additional studies should investigate the movement behaviours of underweight children in western society, who are at elevated risk of adverse health and development delays and tend to be from disadvantaged social backgrounds [55].

Previous studies have reported null associations between parental age with child physical activity [46] and sedentary behaviour [56]. One exception was a study of Canadian children, in whom the prevalence of performing ≥60 min of MVPA per day was lower in 5y old children whose mothers were older at delivery [49]. In both ethnic groups, we observed that children with older mothers tended to be more sedentary and less physically active. Beyond childhood there is an age-related decline in physical activity [42] and in young children maternal-child activity levels are closely related [28, 57]. For these reasons, it is conceivable that lower physical activity and higher sedentary time performed by older mothers may have translated to more of the same behaviours in their children. There might also be biological consequences of having an older mother that predispose to suboptimal movement profiles [58]. For instance, children born to women aged ≥30y are at elevated risk of prematurity, which appears to predict decreased lung function [59] and delayed motor development [60]. Birth weight is also lower in children born to older mothers, and lower birth weight is associated with childhood morbidity [61], and diminished aerobic and neuromuscular fitness [62]. This may explain our observation that South Asian children of high birth weight performed more MVPA and registered more vector magnitude CPM than their normal birth weight peers. We observed no comparable associations in white British children. This apparent ethnic difference could be because South Asian children are typically smaller at birth [63] and hence a threshold of > 3500 g constituted a more extreme birth weight for South Asian than white British children. A recent best evidence synthesis concluded that there is limited evidence that birth weight predicts movement behaviours, with the exception of extreme birth weights predicting later physical activity [64].

#### Prevalence and factors associated with physical inactivity

Nearly all valid days were characterised by ≥180 min of total physical activity at any intensity, which until recently was the recommendation for all children younger than 5y. However, on two-thirds of days children were deemed to be inactive, as they did not satisfy the new international guideline daily amount of physical activity for 3 to 4y olds (≥180 min of total physical activity, including ≥60 min MVPA [3–7]). There were no significant ethnic differences in the likelihood of meeting guidelines, but there was some indication that white British boys were more likely to be inactive compared to South Asian boys. In both ethnic groups, every additional year of age was associated with lower odds of physical inactivity. South Asian children were less likely to be physically inactive on weekends compared to weekdays, and relative to winter they were half as likely to be physically inactive in all other seasons. White British children were less likely to be physically inactive in spring than winter. By far the strongest association was for South Asian girls, who were 3.5 times more likely to be physically inactive compared to South Asian boys. Our results highlight that certain periods may be better targets for reducing the prevalence of childhood inactivity (e.g. focussing on South Asian children on weekdays and in winter) and that targeting children from a young age may pay dividends. South Asian girls appear to have the most to gain from a physical activity intervention.

#### Strengths & weaknesses

Data were harmonised from three studies to permit investigation of a relatively large sample of South Asian and white British children from a materially deprived urban location. This is an important population to study as it is high risk for childhood obesity and subsequent adverse health, and can aid understanding of ethnic inequalities [65]. It is unfortunate that due to small numbers all children of South Asian origin were considered as one group. This precluded a more detailed description of movement behaviours by specific countries of origin. It is also unfortunate that there were substantial missing data for annual income and birth weight; although our analysis incorporated multiply imputed data, the results for these specific parameters should be considered exploratory and deserving of additional research attention. Triaxial accelerometry and a short sampling interval were advantageously used to investigate the whole range of movement behaviours, including light physical activity, for which the evidence of health benefits continue to emerge [1]. Combining the accelerometer data with contextual information to explain patterns of movement behaviours, and to identify the types of sedentary behaviour performed by children, would have been beneficial [66]. Nonetheless, this is the first study to quantify levels of movement behaviours in a young group of bi-ethnic children from a deprived location, and to investigate patterns of movement by numerous socio-demographic, temporal and perinatal characteristics. The prevalence of healthy weight, overweight and obesity in this study sample closely matched that of all children aged 4 to 5y in Bradford who were measured in 2017/18 as part of the National Child Measurement Programme [67]. This provides further reassurance that the study sample represented the source population. Our results are likely generalisable to other young South Asian and white British children who are living in materially deprived UK cities.

## Conclusions

South Asian and white British children spend half of daily time sedentary, just over 40% in light physical activity, and remaining time in MVPA. Sedentary time and physical activity levels differ according to child age, time-related factors including type of day and season, and perinatal factors including birth weight and maternal delivery age. South Asian girls are the most likely to be physically inactive, and as such warrant consideration as priority recipients of programmes to increase physical activity levels.

## Data Availability

The datasets generated and analysed for the current study are available from the corresponding author on reasonable request.

## Abbreviations

BMI: Body mass index
CI: Confidence interval
CPM: Counts per minute
MVPA: Moderate-to-vigorous physical activity
OR: Odds ratio
RCT: Randomised control trial
